# Socioeconomic determinants and inequalities in the prevalence of non-communicable diseases in Saudi Arabia

**DOI:** 10.1186/s12939-021-01510-6

**Published:** 2021-07-28

**Authors:** Mohammed Khaled Al-Hanawi

**Affiliations:** 1 Department of Health Services and Hospital Administration, Faculty of Economics and Administration, King Abdulaziz University, Jeddah, Saudi Arabia; 2 Health Economics Research Group, King Abdulaziz University, Jeddah, Saudi Arabia

**Keywords:** Inequalities, Non-communicable diseases, Public health, Saudi Arabia, Socio-economic status

## Abstract

**Background:**

Non-communicable diseases (NCDs) are increasingly becoming a challenge worldwide, causing high mortality and morbidity. Saudi Arabia has one of the highest rates of NCDs globally and the highest in the Arabian Gulf region. Epidemiological data indicate that NCDs are responsible for 70 % of all deaths in Saudi Arabia. The aim of this study was to examine the socioeconomic determinants and inequalities in the prevalence of NCDs in Saudi Arabia.

**Methods:**

Data from the Saudi Family Health Survey conducted in 2018 by the General Authority for Statistics were used for this study. Univariate, bivariate, and multivariate logistic regression analyses were employed to examine the socioeconomic factors associated with the prevalence of NCDs. Moreover, the concentration curve and concentration indices were used to assess inequalities in the prevalence of NCDs.

**Results:**

Among the 11,527 respondents, the prevalence of NCDs was 32.15 %. The prevalence of NCDs was higher among women and among elderly respondents aged ≥ 60 years. With respect to the determinants of the prevalence of NCDs, the logistic regression results showed that the likelihood of reporting NCDs was lower among people with a higher education (OR: 0.599, 95 % CI: 0.497–0.723, *p < *0.01) compared with that of people with an education below the primary school level. Other factors significantly associated with the prevalence of NCDs were age, marital status, nationality, and region of residence. The inequality analysis showed that at the national level, the prevalence of NCDs was concentrated among less educated people (concentration index = − 0.338, *p* < 0.01), but with significant regional variations. Gender disaggregation showed that both income-based and education-based concentration indices were significantly negative among women, indicating that the prevalence of NCDs is concentrated among women with a lower income level and with less education.

**Conclusions:**

The findings of this study are important for policymakers to combat both the increasing prevalence of and socio-economic inequalities in NCDs. The government should develop targeted intervention strategies to control NCDs and achieve health equality considering socio-economic status. Future policies should target women and the lower educated population in Saudi Arabia.

**Supplementary Information:**

The online version contains supplementary material available at 10.1186/s12939-021-01510-6.

## Introduction

Over the past few decades, the epidemiological progression of diseases has shifted from predominantly communicable diseases towards non-communicable diseases (NCDs) [1]. NCDs, also known as chronic diseases, tend to be of long duration and are the result of a combination of genetic, physiological, behavioural, and environmental factors. This shift to a predominance of NCDs is also attributable to demographic, economic, and environmental changes over time [2]. These changes include the effects of increasing tobacco and alcohol use, physical inactivity, and unhealthy habits, which are the main risk factors associated with the rapid rise in NCDs [3].

The burden of NCDs remains a global public health challenge, leading to high mortality and morbidity. Constituting chronic illnesses that are not infectious or contagious in nature, NCDs are the largest cause of premature death worldwide [4]. According to the World Health Organization (WHO), NCDs kill approximately 41 million people annually, accounting for 71 % of all deaths globally [5]. The main types of NCDs are cardiovascular diseases, chronic respiratory diseases, hypertension, diabetes, and cancers.

Global demographic data suggest that the global population has evolved tremendously. NCDs have spread from the Western hemisphere to global populations. Notably, countries in the Middle East, including the Kingdom of Saudi Arabia (KSA), have experienced this transition [6]. An analysis of the proportional distribution of causes of mortality in 1990–2010 and 2010–2017 in the KSA revealed that NCDs remained relatively stable in both time periods. Even though cardiovascular mortality increased between 1990 and 2010, it remained roughly stable between 2010 and 2017 [6]. However, a review of the wider literature revealed that although the proportional cause of death of NCDs has remained stable in the KSA, the prevalence of NCDs has actually increased.

One study found that the prevalence of hypertension is increasing in the KSA, affecting 26.1 % of the adult population [7]. Other NCDs such as metabolic syndrome, diabetes mellitus, obesity, and dyslipidaemia are also on the rise in the KSA [8, 9]. A recent study showed that increasing physical inactivity and maintenance of an unhealthy diet have led to an increase in the prevalence of these NCDs in the KSA [10]. Evidence suggests that the prevalence of diabetes mellitus, hypertension, and dyslipidaemia in the KSA increased from 9.7 %, 11.4 %, and 9 % in the 1970 s to 26.2 %, 28.6 %, and 18.6 %, respectively, in 2000–2009 among men [11].

There are two main reasons for the increased prevalence of NCDs in the KSA. First, there has been an increasing trend towards unhealthy lifestyles and harmful health behaviours, which may explain the changes in the disease patterns that have shifted from predominantly communicable diseases towards NCDs [12]. Second, there has been an improvement in human longevity in the KSA, as evidenced by an increase in life expectancy from 64 years in the 1980 s to 74 years in the 2000 s [13]. This increase in life expectancy has caused a tandem increase in the prevalence of NCDs, which rises with age [14].

The effects of NCDs are largely prolonged, thereby requiring long-term care and attention. Unlike other diseases with a substantial burden in developing countries, NCDs are a challenge to low-, middle-, and high-income countries alike [15]. Aside from premature death, NCDs undermine social and economic development [16]. The potential of NCDs to reduce quality of life and alter healthcare costs highlights the need to institute operational prevention and control mechanisms. Furthermore, NCDs exacerbate the inequality gaps in health relating to people’s health condition and healthcare access. Inequality in the prevalence of NCDs is one of the leading causes of inequality in life expectancy and mortality [17]. This inequality has attracted substantial attention from several researchers.

In particular, there has been extensive research on the inequalities in the prevalence of NCDs in low-, middle-, and high-income countries [15, 18]. However, there is a scarcity of literature in the KSA regarding the socioeconomic inequalities in the prevalence of NCDs. Previous studies on NCDs have mainly focused on assessing prevalence rates, without taking into account the dimension of income and education-related inequalities, which are crucial factors for policy intervention. Additionally, the bulk of available studies have analysed specific types of chronic NCDs and did not use nationally representative data owing to the scarcity of such data.

To the best of the author’s knowledge, there is no study from the KSA that has analysed the socio-economic inequalities in the prevalence of NCDs. Specifically, no study has used concentration indices to examine the inequality dimensions of NCDs. Therefore, the aim of this study was to fill this gap in the literature by employing univariate, bivariate, and multivariate logistic regression analyses to examine the socioeconomic factors associated with the prevalence of NCDs in the KSA. Moreover, the socio-economic inequalities in the prevalence of NCDs were determined by employing concentration indices and concentration curves. Unlike most of the aforementioned studies, this study used a recent rich dataset that has national representativeness and focused on economic consequences, thereby providing useful guidance on building effective health policies and interventions to curtail the burden of NCDs in the KSA.

The KSA is a compelling case to examine these types of socio-economic inequalities on several grounds. Firstly, Saudi Arabia has one of the highest rates of NCDs in the world and the highest in the Arabian Gulf region. Epidemiological data indicate that NCDs are responsible for 70 % of all deaths in the KSA [19]. Secondly, inherent inequalities between men and women, and across regions in the country make the KSA an interesting case to examine the prevalence of NCDs on such inequalities. Lastly, the healthcare system in Saudi Arabia occupies a large share of the national budget and is undergoing improvement in line with Saudi Vision 2030. Accordingly, this study is both relevant and timely for the conceptualization and implementation of adjustments to the healthcare system of the KSA.

## Materials and methods

### Study setting

This study was conducted in the KSA, the largest country in the Middle East with a land size of approximately 2,150,000 km^2^. The KSA is one of the largest oil producer and exporter countries in the world, and is one of the most important Islamic heritage sites [20]. The KSA relies heavily on oil revenues for its economy, which are used to finance most public sectors, including the healthcare sector [21]. According to the World Bank, the KSA is classified as a high-income country with a high Human Development Index [22]. The KSA also has one of the youngest populations in the world, with 19.6 million people under the age of 30, representing about 57 % of the total population [23].

### Data

Self-weighted data from the Saudi Family Health Survey (FHS) conducted in 2018 by the General Authority for Statistics (GaStat) [24] were used in this study. The FHS is the first collaborative stage between GaStat and several entities in the health sector in the Kingdom, such as the Ministry of Health, the Saudi Health Council, as well as the private and academic sectors. The FHS is a field survey conducted by GaStat every three years, which falls under the classification of education and health statistics. The FHS collects information by visiting a representative sample of the population across all administrative regions in the KSA.

The survey questionnaire was drafted and designed by health statistics experts at GaStat. International recommendations, standards, and definitions issued by the WHO were taken into consideration during the design of the questionnaire. The survey contains several questions to collect information relating to geographical data, basic information of household members, marriage and family planning data, fertility and mortality data, family income and expenditure, and health status of individuals, including if they suffer from any chronic diseases, among other topics [24]. The FHS collected a total sample of 15,265 responses randomly selected across all 13 regions of the Kingdom. For this study, the analysis was limited to respondents who provided complete information on all variables of interest. Therefore, this analysis was based on a sample of 11,527 respondents.

### Measurements

The FHS collected information on the health status of individuals, including a question asking if they suffer from any chronic diseases. This question was given a value of 1 for a response of “yes” and 0 for a response of “no”. This binary variable was then used as the dependent variable for examining the socio-economic determinants and inequalities in the prevalence of chronic NCDs in the KSA.

Other socio-economic and demographic characteristics, including age, gender, marital status, nationality, education level, monthly income, and region of residence, were used as independent variables, with income and education level used as the socio-economic status indicators among the respondents. The age variable was divided into five categories: 18 to 29 (reference category), 30 to 39, 40 to 49, 50 to 59, and ≥ 60 years. Gender was assigned a value of 1 if the respondent was a man and 0 if the respondent was a woman. Marital status was also captured as a binary variable, with a value of 1 given for married respondents and 0 for unmarried respondents (including never married, divorced, and widowed). Nationality was given a value of 1 if the respondent was Saudi and 0 if non-Saudi. Education level was grouped as follows: below primary school (reference), primary school, intermediate school, high school, and higher education. Monthly income [in Saudi Riyal (SR); 1 SR = USD 0.27] was grouped into eight categories: less than SR 3000 (reference category), SR 3000 to < 5000, SR 5000 to < 7000, SR 7000 to < 10,000, SR 10,000 to < 15,000, SR 15,000 to < 20,000, SR 20,000 to < 30,000, and SR 30,000 or more. To account for regional differences, the region variable was grouped into the 13 administrative regions: Riyadh (reference), Albaha, Aljouf, Aseer, Eastern Region, Haiel, Jazan, Madenah, Mekkah, Najran, Northern Borders, Qaseem, and Tabouk.

### Statistical analysis

Univariate analysis was first employed to estimate the percentages and frequencies of respondents for the characteristics of interest. Bivariate analysis was also employed to cross-tabulate the dependent variable and measure the associated frequencies using a Chi-squared test. Multivariate logistic regression models were estimated to examine the socio-economic factors associated with the prevalence of NCDs. Different models were estimated after controlling for age, gender, marital status, nationality, and region of residence. Given that covariate measurement error via misclassification of categorical independent variables in the regression analysis may potentially pose a threat to the validity of parameter estimates and statistical inferences, the study conducted a sensitivity analysis via re-estimation by bootstrap and jackknife approaches [25]. Additionally, the methodology of Wagstaff et al. [26] was adopted to measure socio-economic inequalities in the prevalence of NCDs. This includes visualisation and estimation of inequalities using the concentration curve and the concentration index. Moreover, apart from understanding the general inequality at the national level, the study measured gender and regional inequalities in the prevalence of NCDs which are the common variables by which disparities in health occur as per literature [9, 27–29].

The concentration curve plots the cumulative percentage of a health variable on the vertical axis against the cumulative share of that variable in the population (ranked from the lowest to the highest by an indicator of the socio-economic status) on the horizontal axis. A concentration curve above the line of equality indicates that the prevalence of NCDs is concentrated among the poor, whereas a concentration curve below the line of equality indicates that the prevalence of NCDs is concentrated among the rich. Similarly, with respect to education, a concentration curve above the line of equality indicates that the prevalence of NCDs is concentrated among the less educated and a concentration curve below the line of equality indicates that the prevalence of NCDs is concentrated among the well-educated people. The further the concentration curve diverges from the line of equality (i.e. the 45-degree line), the greater the degree of inequality. The concentration index was calculated as twice the area between the concentration curve and the line of equality to quantify the degree of socio-economic-related inequality in a health sector variable [30]. The concentration index ranges between − 1 and + 1, whereby a positive index indicates that the prevalence of NCDs is disproportionately concentrated among the rich and a negative index indicates concentration among the poor. Similarly, with respect to education, a negative concentration index indicates that the prevalence of NCDs is concentrated among the less educated and a positive concentration index indicates concentration among the well-educated people.

### Ethical clearance

This study was based on the use of secondary data from the FHS, which was conducted, commissioned, funded, and managed in 2018 by GaStat that was in charge of all ethical procedures. All procedures performed in this study involving human participants complied with the institutional and/or national research committee ethical standards, and with the 1964 Helsinki Declaration and subsequent amendments or equivalent ethical standards. Informed consent was obtained from all participants. All personal identifiers were removed from the dataset by GaStat to allow for secondary data use. GaStat granted permission to use the data and thus no further clearance was necessary as this was performed at the data collection phase.

## Results

### Descriptive statistics

Among the total sample of 11,527 respondents, 32.15 % reported suffering from NCDs. Approximately one-third of the sample was in the age range of 18–29 years, 55 % were men, and two-thirds of the sample were married. With respect to education level, 19.32 % of the respondents had no schooling or below primary school education and 19.88 % completed higher education. Table 1 shows the full characteristics of the sample.

**Table 1:** Descriptive statistics of the sample (*N* = 11,527)

Variable	Frequency	%
**Prevalence of NCDs**	3706	32.15
**Age**		
18–29	3733	32.38
30–39	2398	20.80
40–49	1805	15.67
50–59	1569	13.61
≥ 60	2022	17.54
**Gender**		
Female	5269	45.71
Male	6258	54.29
**Marital status**		
Married	7395	64.15
Unmarried	4132	35.85
**Education level**		
Below primary school	2226	19.32
Primary school	1246	10.81
Intermediate school	1893	16.42
Secondary school	3870	33.57
Higher education	2292	19.88
**Nationality**		
Non-Saudi	2882	25.00
Saudi	8645	75.00
**Monthly income (Saudi Riyal)**		
< 3000	1062	9.21
3000 to < 5000	1851	16.06
5000 to < 7000	1777	15.42
7000 to < 10,000	2220	19.26
10,000 to < 15,000	2173	18.85
15,000 to < 20,000	1118	9.70
20,000 to < 30,000	721	6.25
≥ 30,000	605	5.25
**Region**		
Riyadh	1652	14.33
Albaha	745	6.46
Aljouf	386	3.35
Aseer	581	5.05
Eastern Region	1049	9.10
Haiel	745	6.46
Jazan	663	5.75
Madenah	834	7.23
Mekkah	2003	17.38
Najran	408	3.54
Northern Border	411	3.57
Qassim	1261	10.94
Tabuk	789	6.84

### Bivariate analysis

Table 2 shows the results of bivariate analysis of the association between the prevalence of NCDs and socio-economic characteristics. The prevalence of NCDs was significantly associated with gender (χ^2^ = 10.51, *p* < 0.01), in which NCDs were more concentrated among women (33.68 %) than men (30.86 %). There were significant associations between the prevalence of NCDs and marital status (χ^2^ = 404.21, *p* < 0.01) and region of residence (χ^2^ = 274.39, *p* < 0.01). Moreover, there was a significant association between the prevalence of NCDs and educational attainment (χ^2^ = 160.63, *p* < 0.01). Compared with that of highly educated people (20.07 %) and those with secondary school education (17.98 %), the prevalence of NCDs was much higher among people with no schooling or with a below primary school level of education (64.11 %). Moreover, the prevalence of NCDs was significantly associated with nationality (χ^2^ = 275.76, *p* < 0.01), in which the prevalence of chronic diseases was more heavily concentrated among Saudis (36.32 %) than non-Saudis (19.64 %).

**Table 2:** Bivariate analysis of the prevalence of NCDs and socio-economic characteristics (*N* = 11,527)

Variable	Frequency	Percent with NCDs	Chi square
**Age**			520.23***
18–29	3733	5.04	
30–39	2398	9.01	
40–49	1805	31.80	
50–59	1569	63.10	
≥ 60	2022	85.95	
**Gender**			10.51***
Female	5269	33.68	
Male	6258	30.86	
**Marital status**			404.21***
Married	7395	38.69	
Unmarried	4132	20.45	
**Education level**			160.63***
Below primary school	2226	64.11	
Primary school	1246	43.82	
Intermediate school	1893	30.48	
Secondary school	3870	17.98	
Higher education	2292	20.07	
**Nationality**			275.76***
Non-Saudi	2882	19.64	
Saudi	8645	36.32	
**Monthly income (Saudi Riyal)**			88.26***
< 3000	1062	30.32	
3000 to < 5000	1851	33.87	
5000 to < 7000	1777	33.99	
7000 to < 10,000	2220	30.86	
10,000 to < 15,000	2173	30.00	
15,000 to < 20,000	1118	30.14	
20,000 to < 30,000	721	26.77	
≥ 30,000	605	47.27	
**Region**			274.39***
Riyadh	1,652	32.26	
Albaha	745	38.66	
Aljouf	386	24.09	
Aseer	581	29.78	
Eastern Region	1,049	36.42	
Haiel	745	25.91	
Jazan	663	17.95	
Madenah	834	30.58	
Mekkah	2,003	34.30	
Najran	408	20.83	
Northern Border	411	14.60	
Qassim	1,261	42.66	
Tabuk	789	38.02	

*** *p* < 0.01, ** *p* < 0.05, * *p* < 0.1

To determine the level of inequalities in the prevalence of NCDs in various socioeconomic groups (i.e. income and education), the Wagstaff inequality concentration index was estimated. The results are presented in Table 3.

**Table 3:** Wagstaff inequality indices for the prevalence of NCDs by income and education (*N* = 11,527)

	Income		Education	
	**Index estimate**	**95 % CI**	**Index estimate**	**95 % CI**
**National level**	0.003	(–0.020 to 0.025)	–0.338***	(–0.358 to − 0.316)
**Gender**				
Female	–0.128***	(–0.160 to − 0.095)	–0.513***	(–0.541 to 0.484)
Male	0.113***	(0.083 to 0.144)	–0.173***	(–0.203 to − 0.144)
**Regions**				
Riyadh	0.002	(–0.056 to 0.061)	–0.202***	(–0.259 to − 0.145)
Albaha	–0.098**	(–0.181 to − 0.015)	–0.434***	(–0.511 to − 0.357)
Aljouf	–0.123*	(–0.253 to 0.009)	–0.298***	(–0.425 to − 0.171)
Aseer	0.049	(–0.053 to 0.151)	–0.352***	(–0.446 to − 0.253)
Eastern Region	–0.044	(–0.115 to 0.028)	–0.310***	(–0.377 to − 0.243)
Haiel	0.095**	(0.001 to 0.188)	–0.542***	(–0.625 to 0.459)
Jazan	–0.084	(–0.196 to 0.029)	–0.412***	(–0.518 to − 0.305)
Madenah	–0.190***	(–0.272 to − 0.107)	–0.585***	(–0.658 to − 0.513)
Mekkah	–0.076***	(–0.128 to 0.024)	–0.276***	(–0.326 to − 0.225)
Najran	–0.112	(–0.248 to 0.024)	–0.550***	(–0.672 to − 0.428)
Northern Border	0.333***	(0.180 to 0.484)	–0.253***	(–0.404 to 0.102)
Qassim	0.147***	(0.083 to 0.209)	–0.233***	(–0.294 to − 0.172)
Tabuk	–0.122***	(–0.203 to − 0.041)	–0.611***	(–0.679 to − 0.542)

*** *p* < 0.01, ** *p* < 0.05, * *p* < 0.1

At the national level, the education-based concentration index was − 0.338, which was statistically significant at the 1 % level, demonstrating that the overall prevalence of NCDs is concentrated among the less educated people in the KSA. By gender, both income-based and education-based concentration indices were statistically significant at the 1 % level. Among women, both the income-based and education-based indices were significantly negative, indicating that the prevalence of NCDs is concentrated among women with a lower income level and with less education. By contrast, the income-based concentration index among men was significantly positive, indicating that the prevalence of NCDs is concentrated among men with a higher income level. 

Although the education-based concentration indices were negative and statistically significant at the 1 % level for all regions, this was not the case for the income-based concentration indices across regions. For some regions, the inequality indices were either not significantly negative or were significantly positive. For instance, the income-based concentration indices in Hail (0.095, *p* < 0.05), Northern Border (0.333, *p* < 0.01), and Qassim (0.147, *p* < 0.01) were positive and statistically significant. In Riyadh and Aseer, the income-based indices were also positive but were not statistically significant, whereas the negative income-based indices in the Eastern Region, Jazan, and Najran were statistically insignificant.

**Fig. 1:**
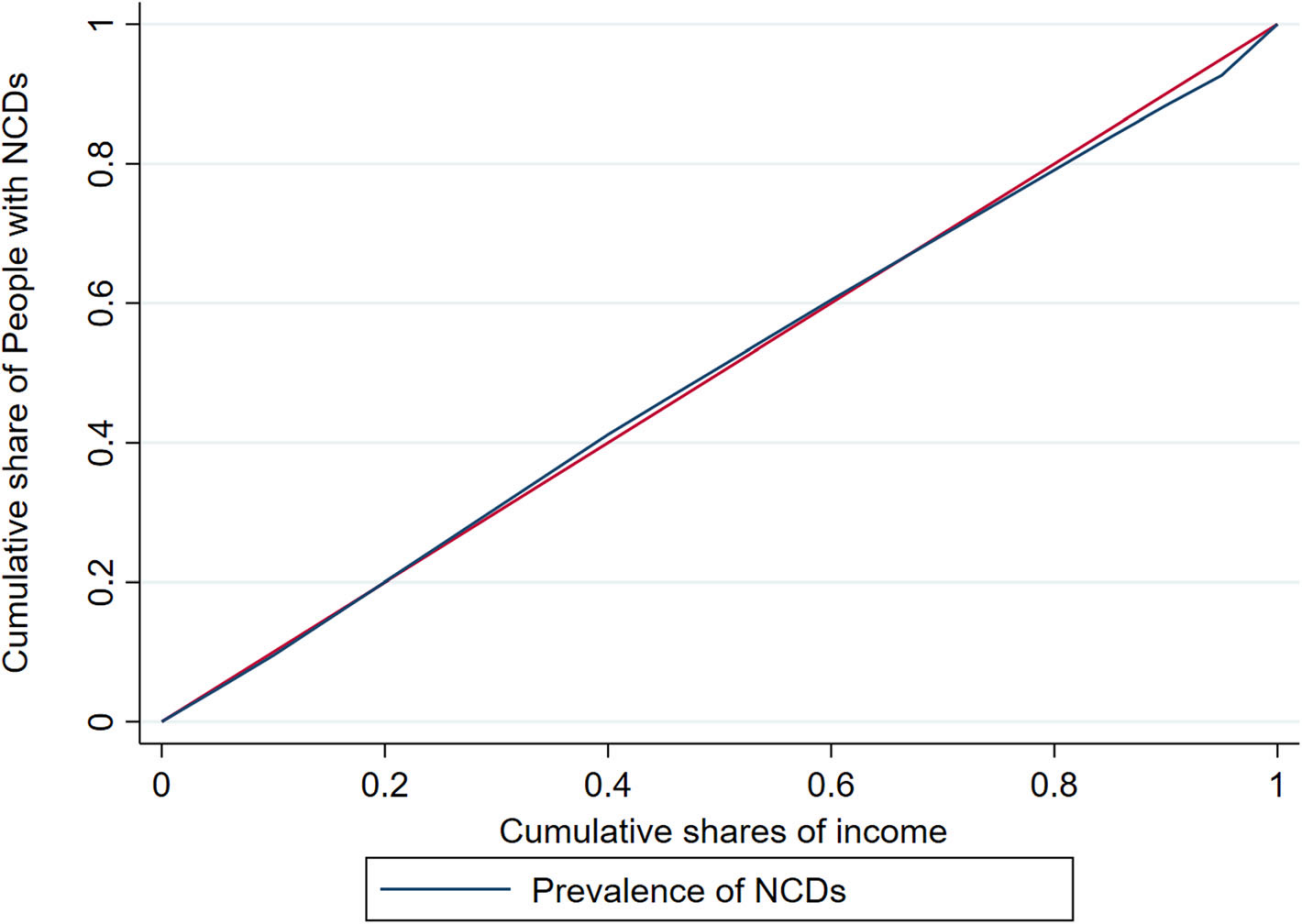
Income-based concentration curve

**Fig. 2:**
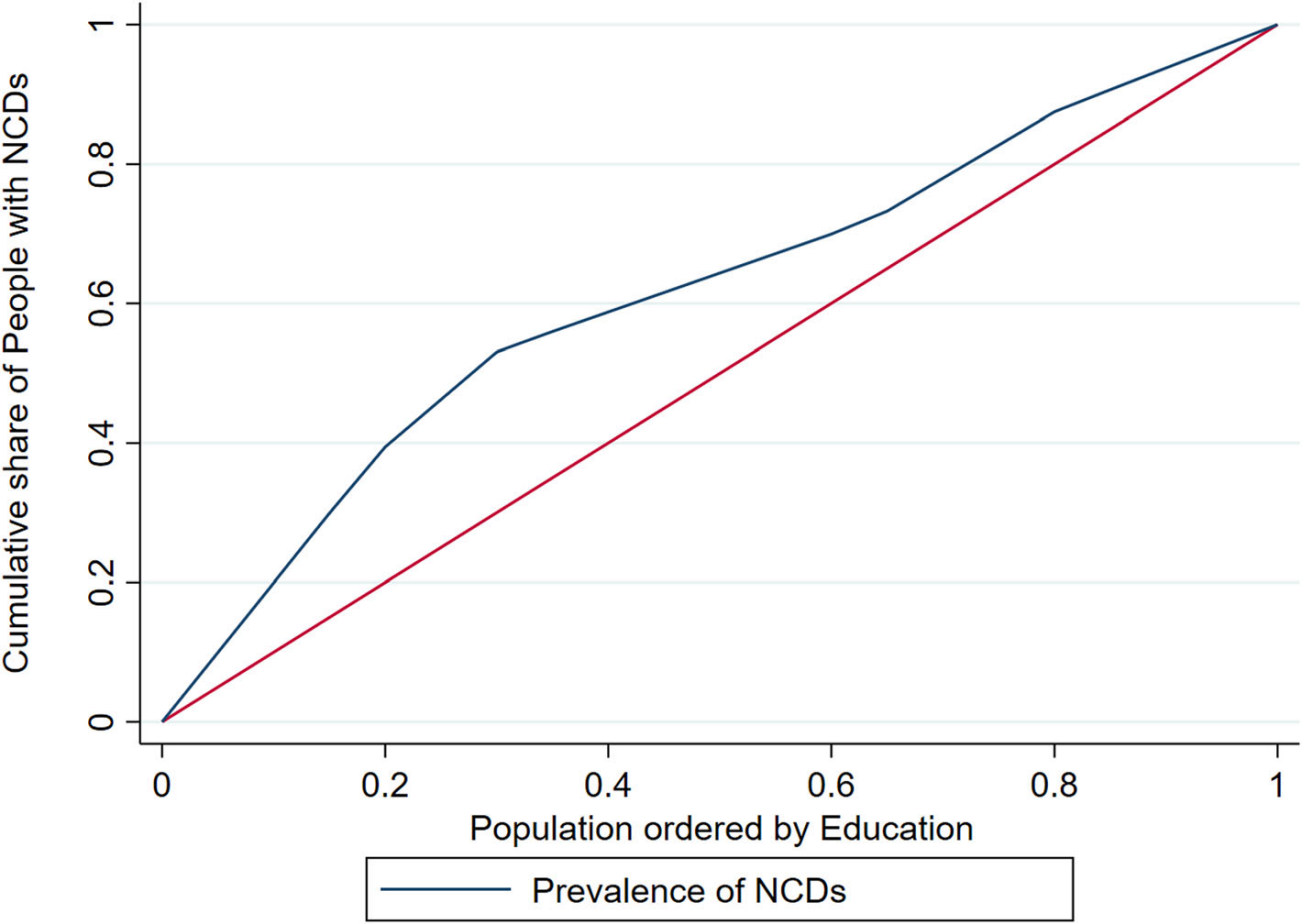
Education-based concentration curve

Figures 1 and 2 depict the concentration curves by income and education, respectively. Figure 1 shows that the income-based concentration curve for people with NCDs almost perfectly overlaps with the line of equality, suggesting no inequality. By contrast, Fig. 2 confirms that the prevalence of NCDs is disproportionately concentrated among less educated people in the KSA, as the concentration curve (blue) is above the red line of equality.

### Regression analysis

Given that the univariate and bivariate analyses did not consider other variables that might influence the association between the prevalence of NCDs and socio-economic factors, a multivariate logistic regression analysis was further performed. Table 4 summarises the results from the logistic regression analysis to examine the socio-economic factors associated with the prevalence of NCDs.

**Table 4:** Association between the prevalence of NCDs and socio-economic factors (logistic regression)

Variables	Model 1		Model 2		Model 3	
	**OR**	**95 % CI**	**OR**	**95 % CI**	**OR**	**95 % CI**
**Age**						
18–29	Reference		Reference		Reference	
30–39	2.662***	(2.123–3.337)	2.761***	(2.194–3.475)	2.764***	(2.196–3.478)
40–49	13.069***	(10.510–16.250)	12.422***	(9.929–15.542)	12.537***	(10.019–15.689)
50–59	46.303***	(37.219–57.603)	42.104***	(33.521–52.885)	42.423***	(33.764–53.303)
≥ 60	149.848***	(119.820-187.401)	125.850***	(98.683-160.496)	126.621***	(99.256-161.532)
**Gender**						
Female	Reference		Reference		Reference	
Male	0.849***	(0.759–0.950)	0.924	(0.823–1.038)	0.915	(0.814–1.029)
**Marital status**						
Married	Reference		Reference		Reference	
Unmarried	1.543***	(1.312–1.814)	1.526***	(1.296–1.799)	1.506***	(1.277–1.775)
**Education level**						
Below primary school			Reference		Reference	
Primary school			0.877	(0.723–1.063)	0.900	(0.741–1.093)
Intermediate school			1.055	(0.873–1.275)	1.107	(0.912–1.344)
Secondary school			0.622***	(0.523–0.740)	0.654***	(0.546–0.784)
Higher education			0.599***	(0.497–0.723)	0.650***	(0.531–0.795)
**Nationality**						
Non-Saudi	Reference		Reference		Reference	
Saudi	1.962***	(1.697–2.267)	1.853***	(1.616–2.125)	1.921***	(1.662–2.223)
**Monthly income (Saudi Riyal)**						
< 3000	Reference				Reference	
3000 to < 5000	1.103	(0.875–1.391)			1.131	(0.896–1.427)
5000 to < 7000	1.074	(0.849–1.359)			1.139	(0.899–1.443)
7000 to < 10,000	0.803*	(0.637–1.011)			0.907	(0.716–1.150)
10,000 to < 15,000	0.765**	(0.606–0.966)			0.886	(0.696–1.127)
15,000 to < 20,000	0.729**	(0.561–0.947)			0.868	(0.661-0.1.139)
20,000 to < 30,000	0.629***	(0.469–0.844)			0.760*	(0.560–1.032)
≥ 30,000	1.202	(0.879–1.645)			1.506**	(1.089–2.083)
**Region**						
Riyadh	Reference		Reference		Reference	
Albaha	0.710***	(0.551–0.914)	0.688***	(0.534–0.887)	0.688***	(0.533–0.888)
Aljouf	0.543***	(0.379–0.777)	0.573***	(0.403–0.816)	0.540***	(0.377–0.773)
Aseer	0.708**	(0.531–0.945)	0.688**	(0.515–0.918)	0.664***	(0.497–0.888)
Eastern Region	0.639***	(0.510–0.802)	0.645***	(0.514–0.809)	0.636***	(0.506-0.800)
Haiel	0.545***	(0.416–0.713)	0.565***	(0.432–0.740)	0.527***	(0.402–0.691)
Jazan	0.414***	(0.308–0.556)	0.421***	(0.313–0.565)	0.419***	(0.312–0.563)
Madenah	0.649***	(0.505–0.832)	0.674***	(0.527–0.862)	0.641***	(0.498–0.824)
Mekkah	1.046	(0.865–1.266)	1.069	(0.886–1.291)	1.028	(0.849–1.245)
Najran	0.440***	(0.314–0.616)	0.412***	(0.294–0.578)	0.416***	(0.297–0.584)
Northern border	0.412***	(0.280–0.606)	0.408***	(0.277–0.602)	0.374***	(0.252–0.555)
Qassim	1.263**	(1.023–1.556)	1.330***	(1.080–1.636)	1.243**	(1.006–1.536)
Tabuk	0.797*	(0.619–1.027)	0.783*	(0.608–1.008)	0.765**	(0.592–0.987)
**Constant**	0.033***	(0.024–0.046)	0.040***	(0.029–0.054)	0.039***	(0.027–0.055)
**Observations**	11,527		11,527		11,527	
**Pseudo R-squared**	0.408		0.410		0.411	
**Chi-squared**	5901***		5922***		5954***	

Note. 95 % confidence intervals are in parentheses; Abbreviation: OR, odds ratio; *** *p* < 0.01, ** *p* < 0.05, * *p* < 0.1

The likelihood of reporting NCDs was lower among the higher-income groups (except those who earn ≥ 30,000 SR) compared with the lower income groups, as revealed by Model 1. For example, the odds ratio (OR) was 0.629 (95 % CI: 0.469–0.844, *p* < 0.01) for people with a reported income level of 20,000 to < 30,000 SR. Model 2 indicated that the likelihood of reporting NCDs was lower among people with a higher education (OR: 0.599, 95 % CI: 0.497–0.723, *p* < 0.01) compared with that of people with an education below the primary school level. The OR of education categories remained statistically significant in Model 3. Model 3 also revealed the higher likelihood of reporting NCDs among people aged ≥ 60 years (OR: 126.62, 95 % CI: 99.256-161.532, *p* < 0.01) compared with that of younger people.

In addition, the likelihood of reporting NCDs among unmarried people was higher compared with that of married people (OR: 1.51, 95 % CI: 1.277–1.775, *p* < 0.01). Moreover, the OR for the regional dummy variable suggested that there are regional differences in the likelihood of reporting NCDs. Specifically, residents in most of regions, except for Qassim (OR: 1.243, 95 % CI: 1.006–1.536, *p* < 0.05), were less likely to report NCDs than those in Riyadh.

Given that covariate measurement error via misclassification of categorical independent variables in the regression analysis may potentially pose a threat to the validity of parameter estimates and statistical inferences, the study also conducted a sensitivity analysis via re-estimation by bootstrap and jackknife approaches. The OR findings in the bootstrap and jackknife estimation approaches were consistent with the initial findings (Appendix 1), suggesting that the model was robust to any likely measurement error.

## Discussion

This is the first study to investigate socio-economic determinants and inequalities in the prevalence of NCDs in the KSA using a vast dataset with national representativeness. These characteristics distinguish this study from previous studies that analysed the prevalence of specific types of NCDs among diverse groups [4, 31]. The findings showed that the prevalence of NCDs in the KSA is 32.15 %. Moreover, there were significant inequalities found in the prevalence of NCDs in the KSA across socio-economic characteristics. Aside from health repercussions, the high rate of the prevalence of NCDs and the existence of inequalities have adverse effects on the economy. NCDs affect the quality of health of the labour force, thereby leading to reduction in labour productivity [32]. This has a negative impact on the economy as it hinders growth of the gross domestic product (GDP) per capita and places a huge financial burden on the government for health expenditure [33, 34]. In Saudi Arabia, an economic burden analysis showed that economic losses from NCDs are equivalent to 2.8 % of the GDP [35]. Such effects are worse among vulnerable groups that are more susceptible to NCDs. By reducing their economic activity, NCDs have the potential to keep the vulnerable population in a cycle of struggles and in an inescapable poverty trap [36]. Therefore, there is a dire need to implement control and prevention strategies that account for existing socio-economic inequalities in the prevalence of NCDs.

The results showed that the prevalence of NCDs is significantly associated with gender and is more concentrated among women than men. More specifically, the results showed that the prevalence of NCDs is concentrated among women with a lower income level and with less education. These findings correlate with results from other studies [2, 37–39]. Women generally live longer than men due to a higher rate of death by external causes among men [31]. The high life expectancy among women makes them susceptible to NCDs, which are highly prevalent among the aging population. Moreover, women use healthcare services more frequently due to their greater sensitivity to physical symptoms than men [37]. As such, women are more likely to receive a diagnosis than men in self-reported studies. Accordingly, it is important for countries to consider health strategies that address gender-based inequalities in the prevalence of NCDs. In Saudi Arabia, one of the focus areas in the implementation of Vision 2030 is to close the gap between men and women in personal income, education, and labour force participation [19]. Without addressing these challenges, the contribution of women to the economic development of the country would be greatly undermined.

Regarding age, the likelihood of reporting NCDs was higher among elderly people aged ≥ 60 years compared with that of younger people. This is coherent with the literature showing higher occurrences of NCDs among the elderly population [17, 38, 40, 41]. Furthermore, the prevalence of NCDs appears to be concentrated among the less educated people in Saudi Arabia. Contrasting the prevalence for highly educated people (20.07 %) and those with a secondary school level of education (17.98 %), the prevalence of NCDs was 64.11 % among people with no schooling or education below the primary level. The logistic regression also indicated a lower OR of NCDs prevalence among people with higher education (0.599, 95 % CI: 0.497–0.723, p˂0.01) compared with that of people with an education below the primary school level. However, this is not a surprising result as previous studies have shown a high association between a low socio-economic status and high prevalence of NCDs [42].

There were also regional differences found in the likelihood of reporting chronic diseases as shown by the OR for the regional dummy variable. Moreover, the education-based concentration indices were negative and statistically significant at the 1 % level for all regions. This is in line with findings from other studies showing that urban and rural regions exhibited different prevalence levels [18, 40, 43, 44]. These findings are mainly due to disparities in healthcare access and healthcare resources, diverse cultural norms and practices, as well as limited availability of healthcare facilities in different regions of a country [17]. Such regional differences can exacerbate socio-economic inequalities in the prevalence of NCDs, and have the potential to widen the gap between the rich and the poor in the country.

The prevalence of chronic diseases was also significantly associated with nationality. NCDs were found to be more heavily concentrated among Saudis than non-Saudis. The differences in lifestyle between nationals and expatriates might explain this high prevalence rate of NCDs among Saudi nationals, as there is evidence demonstrating that physical inactivity and consumption of an unhealthy diet (risk factors for NCDs) are common and significantly high among adult Saudi citizens [10, 45]. Similar findings were also found in other Arab countries sharing similar customs and traditions [46]. Moreover, this study demonstrated a lower likelihood of reporting chronic disease among higher-income groups (except those earning more than 30,000 SR) compared with that of lower-income groups. A potential explanation for this difference is that at very high levels of income (i.e. those who earn more than 30,000 SR in Saudi Arabia), the prevalence rate of NCDs increases as people become more susceptible to risk factors such as by increasing physical inactivity and unhealthy habits. This result related to the highest income level agrees with results observed in China [47].

This study has several advantages. The analysis involved a rich dataset with a nationally representative sample. This study also applied multiple techniques to reduce method-based findings and conclusions. Therefore, the findings are relevant for informing the design and implementation of control and prevention mechanism for NCDs. Nevertheless, there are a few limitations associated with this study. The data utilized are subject to recall biases as they are self-reported. Future analyses should consider using different types of data or applying standardized measures that could reduce the effect of self-reported data. In addition, disparities in the reported prevalence of NCDs could reflect differential access to healthcare services. There might be higher reports from people with access to a diagnosis than those lacking such access. In this case, the results might be capturing access to diagnosis instead of the true existence of NCDs. This is because the absence of self-reported prevalence might indicate absence of a diagnosis rather than the absence of NCDs. These are some of the issues that future studies need to consider when conducting similar analyses.

## Conclusions

Using data from the 2018 Saudi FHS, this study examined the socio-economic determinants and inequalities in the prevalence of NCDs in the KSA. The general prevalence of NCDs was found to be 32.15 %, and the results established the existence of significant inequalities in the prevalence of NCDs in the KSA. The prevalence of NCDs was higher among women and among elderly people aged 60 years and above. The likelihood of reporting NCDs was also higher among people with lower education levels. These inequalities have been shown to have adverse health as well as economic effects, as they have the potential to derail economic development. There is a need to monitor the progression of risk factors and develop targeted intervention strategies to control NCDs and achieve health equality. Such control and prevention mechanisms must consider socio-economic status, as well as factors such as age, marital status, nationality, and region of residence. This is a prerequisite if such strategies are to be effective in addressing the challenges of rapid population ageing, socio-economic inequalities, and negative economic effects.

## Supplementary information

Additional file 1 Appendix 1: A sensitivity analysis via re-estimation by bootstrap and jackknife approaches.

## Data Availability

The datasets generated and/or analysed during the current study are not publicly available due to privacy, confidentiality, and other restrictions. Access to data can be gained through the General Authority for Statistics in Saudi Arabia via https://www.stats.gov.sa/en.
