# Supplementary material for: Socioeconomic determinants and inequalities in the prevalence of non-communicable diseases in Saudi Arabia
Source: Int J Equity Health. 2021 Jul 28;20:174. doi: 10.1186/s12939-021-01510-6 (PMC8320210; doi:10.1186/s12939-021-01510-6)
Supplement: Supplementary file 1 — Appendix 1: A sensitivity analysis via re-estimation by bootstrap and jackknife approaches. [file 12939_2021_1510_MOESM1_ESM.docx]

Appendix 1: A sensitivity analysis via re-estimation by bootstrap and jackknife approaches

| **Variables** | **Model 1** | | | | **Model 2** | | | | **Model 3** | | | |
| --- | --- | --- | --- | --- | --- | --- | --- | --- | --- | --- | --- | --- |
|  | **Bootstrap Approach** | | **Jackknife Approach** | | **Bootstrap Approach** | | **Jackknife Approach** | | **Bootstrap Approach** | | **Jackknife Approach** | |
|  | **OR** | **95% CI** | **OR** | **95% CI** | **OR** | **95% CI** | **OR** | **95% CI** | **OR** | **95% CI** | **OR** | **95% CI** |
| **Age** |  |  |  |  |  |  |  |  |  |  |  |  |
| 18-29 | (Reference) |  | (Reference) |  | Reference |  | Reference |  | Reference |  | Reference |  |
| 30-39 | 2.662*** | (2.113-3.352) | 2.662*** | (2.113-3.330) | 2.761*** | (2.134-3.574) | 2.761*** | (2.192-3.479) | 2.764*** | (2.167-3.525) | 2.764*** | (2.159-3.479) |
| 40-49 | 13.069*** | (10.603-16.108) | 13.069*** | (10.530-16.220) | 12.422*** | (9.882-15.615) | 12.422*** | (9.912-15.567) | 12.537*** | (9.780-16.072) | 12.537*** | (10.010-15.703) |
| 50-59 | 46.303*** | (38.565-55.593) | 46.303*** | (37.345-57.409) | 42.104*** | (33.415-53.053) | 42.104*** | (33.514-52.897) | 42.423*** | (33.672-53.449) | 42.423*** | (33.767-53.299) |
| ≥60 | 149.848*** | (120.164-186.866) | 149.848*** | (120.09-186.968) | 125.850*** | (98.124-161.410) | 125.850*** | (98.641-160.565) | 126.621*** | (101.52-157.926) | 126.621*** | (99.131-161.74) |
| **Gender** |  |  |  |  |  |  |  |  |  |  |  |  |
| Female | Reference |  | Reference |  | Reference |  | Reference |  | Reference |  | Reference |  |
| Male | 0.849*** | (0.751-0.960) | 0.849*** | (0.759-0.950) | 0.924 | (0.821-1.040) | 0.924 | (0.821-1.040) | 0.915 | (0.800-1.047) | 0.915 | (0.812-1.031) |
| **Marital status** |  |  |  |  |  |  |  |  |  |  |  |  |
| Married | Reference |  | Reference |  | Reference |  | Reference |  | Reference |  | Reference |  |
| Unmarried | 1.543*** | (1.350-1.762) | 1.543*** | (1.320-1.803) | 1.526*** | (1.308-1.781) | 1.526*** | (1.301-1.791) | 1.506*** | (1.289-1.759) | 1.506*** | (1.284-1.766) |
| **Education level** |  |  |  |  |  |  |  |  |  |  |  |  |
| Below primary |  |  |  |  | Reference |  | Reference |  | Reference |  | Reference |  |
| Primary school |  |  |  |  | 0.877 | (0.706-1.089) | 0.877 | (0.721-1.067) | 0.900 | (0.748-1.082) | 0.900 | (0.738-1.098) |
| Intermediate school |  |  |  |  | 1.055 | (0.878-1.269) | 1.055 | (0.875-1.273) | 1.107 | (0.917-1.337) | 1.107 | (0.912-1.345) |
| Secondary school |  |  |  |  | 0.622*** | (0.517-0.748) | 0.622*** | (0.522-0.741) | 0.654*** | (0.539-0.795) | 0.654*** | (0.544-0.787) |
| Higher education |  |  |  |  | 0.599*** | (0.511-0.703) | 0.599*** | (0.5498-0.721) | 0.650*** | (0.542-0.778) | 0.650*** | (0.530-0.796) |
| **Nationality** |  |  |  |  |  |  |  |  |  |  |  |  |
| Non-Saudi | Reference |  | Reference |  | Reference |  | Reference |  | Reference |  | Reference |  |
| Saudi | 1.962*** | (1.716-2.242) | 1.962*** | (1.697-2.267) | 1.853*** | (1.640-2.096) | 1.853*** | (1.610-2.132) | 1.921*** | (1.730-2.135) | 1.921*** | (1.663-2.221) |
| **Monthly income (Saudi Riyal)** |  |  |  |  |  |  |  |  |  |  |  |  |
| ˂3000 | Reference |  | Reference |  |  |  |  |  | Reference |  | Reference |  |
| 3000 to ˂5000 | 1.103 | (0.843-1.444) | 1.103 | (0.865-1.406) |  |  |  |  | 1.131 | (0.869-1.471) | 1.131 | (0.887-1.442) |
| 5000 to ˂7000 | 1.074 | (0.839-1.376) | 1.074 | (0.846-1.364) |  |  |  |  | 1.139 | (0.905-1.433) | 1.139 | (0.895-1.450) |
| 7000 to ˂10,000 | 0.803 | (0.614-1.049) | 0.803* | (0.635-1.015) |  |  |  |  | 0.907 | (0.702-1.173) | 0.907 | (0.711-1.157) |
| 10,000 to ˂15,000 | 0.765** | (0.600-0.975) | 0.765** | (0.604-0.968) |  |  |  |  | 0.886 | (0.669-1.172) | 0.886 | (0.694-1.131) |
| 15,000 to ˂20,000 | 0.729** | (0.563-0.944) | 0.729** | (0.557-0.953) |  |  |  |  | 0.868 | (0.656-1.147) | 0.868 | (0.656-1.148) |
| 20,000 to ˂30,000 | 0.629** | (0.437-0.906) | 0.629*** | (0.463-0.855) |  |  |  |  | 0.760* | (0.555-1.040) | 0.760* | (0.552-1.045) |
| ≥30,000 | 1.202 | (0.905-1.597) | 1.202 | (0.896-1.614) |  |  |  |  | 1.506*** | (1.103-2.055) | 1.506*** | (1.110-2.042) |
| **Region** |  |  |  |  |  |  |  |  |  |  |  |  |
| Riyadh | Reference |  | Reference |  | Reference |  | Reference |  | Reference |  | Reference |  |
| Albaha | 0.710*** | (0.568-0.886) | 0.710*** | (0.561-0.898) | 0.688*** | (0.547-0.866) | 0.688*** | (0.542-0.874) | 0.688*** | (0.547-0.865) | 0.688*** | (0.541-0.875) |
| Aljouf | 0.543*** | (0.340-0.735) | 0.543*** | (0.385-0.765) | 0.573*** | (0.440-0.747) | 0.573*** | (0.412-0.799) | 0.540*** | (0.386-0.755) | 0.540*** | (0.384-0.759) |
| Aseer | 0.708** | (0.516-0.972) | 0.708** | (0.511-0.981) | 0.688** | (0.498-0.949) | 0.688** | (0.496-0.952) | 0.664*** | (0.491-0.898) | 0.664** | (0.478-0.922) |
| Eastern Region | 0.639*** | (0.502-0.814) | 0.639*** | (0.511-0.801) | 0.645*** | (0.514-0.808) | 0.645*** | (0.515-0.807) | 0.636*** | (0.505-0.802) | 0.636*** | (0.507-0.799) |
| Haiel | 0.545*** | (0.413-0.718) | 0.545*** | (0.417-0.712) | 0.565*** | (0.434-0.737) | 0.565*** | (0.433-0.738) | 0.527*** | (0.401-0.692) | 0.527*** | (0.402-0.690) |
| Jazan | 0.414*** | (0.294-0.582) | 0.414*** | (0.306-0.559) | 0.421*** | (0.314-0.564) | 0.421*** | (0.313-0.566) | 0.419*** | (0.301-0.584) | 0.419*** | (0.312-0.564) |
| Madenah | 0.649*** | (0.542-0.776) | 0.649*** | (0.513-0.820) | 0.674*** | (0.539-0.843) | 0.674*** | (0.535-0.850) | 0.641*** | (0.493-0.832) | 0.641*** | (0.505-0.812) |
| Mekkah | 1.046 | (0.865-1.265) | 1.046 | (0.861-1.271) | 1.069 | (0.871-1.312) | 1.069 | (0.880-1.299) | 1.028 | (0.856-1.236) | 1.028 | (0.846-1.250) |
| Najran | 0.440*** | (0.295-0.665) | 0.440*** | (0.302-0.640) | 0.412*** | (0.284-0.598) | 0.412*** | (0.284-0.599) | 0.416*** | (0.299-0.580) | 0.416*** | (0.287-0.604) |
| Northern border | 0.412*** | (0.274-0.620) | 0.412*** | (0.281-0.604) | 0.408*** | (0.280-0.596) | 0.408*** | (0.278-0.601) | 0.374*** | (0.251-0.557) | 0.374*** | (0.251-0.556) |
| Qassim | 1.263** | (1.026-1.554) | 1.263** | (1.029-1.549) | 1.330*** | (1.085-1.629) | 1.330*** | (1.088-1.625) | 1.243** | (1.010-1.529) | 1.243** | (1.012-1.527) |
| Tabuk | 0.797* | (0.623-1.020) | 0.797* | (0.629-1.010) | 0.783** | (0.626-0.979) | 0.783** | (0.616-0.995) | 0.765* | (0.574-1.0108) | 0.765** | (0.600-0.974) |
| **Constant** | 0.033*** | (0.025-0.045) | 0.033*** | (0.024-0.046) | 0.040*** | (0.029-0.054) | 0.040*** | (0.029-0.054) | 0.039*** | (0.027-0.055) | 0.039*** | (0.027-0.055) |
| **Observations** | 11,527 |  | 11527 |  | 11,527 |  | 11,527 |  | 11,527 |  | 11,527 |  |
| **Pseudo R-squared** | 0.408 |  | 0.408 |  | 0.410 |  | 0.410 |  | 0.411 |  | 0.411 |  |
| **Chi-squared** | 8179*** |  |  |  | 6230*** |  |  |  | 8981*** |  |  |  |

Note. 95% confidence intervals are in parentheses; Abbreviation: OR, odds ratio; *** p<0.01, ** p<0.05, * p<0.1
